# Evaluation of Level of Essential Elements and Toxic Metal in the Medicinal Plant* Hymenaea martiana* Hayne (Jatobá) Used by Mid-West Population of Brazil

**DOI:** 10.1155/2019/4806068

**Published:** 2019-06-20

**Authors:** Layza S. Rocha, Daniela G. Arakaki, Danielle Bogo, Elaine S. P. Melo, Nayara V. Lima, Igor D. de Souza, Anthony J. Garrison-Engbrecht, Rita de Cassia A. Guimarães, Valter A. Nascimento

**Affiliations:** ^1^Group of Spectroscopy and Bioinformatics Applied Biodiversity and Health (GEBABS), Federal University of Mato Grosso do Sul, 549, Campo Grande 790709-00, MS, School of Medicine of Federal University of Mato Grosso do Sul, Brazil; ^2^Departamento de Tecnologia de Alimentos e Saúde Pública (DTA), Universidade Federal do Mato Grosso do Sul, Campo Grande/MS, 790709-00, Brasil, 549, Campo Grande, Brazil; ^3^Rural Education, Oregon State University and Leadership, Engagement, Advising, and Development (LEAD) Center at the University of California, Berkeley, USA

## Abstract

*Hymenaea martiana *Hayne belongs to the family* Fabaceae* (*Leguminosae*) and presents the popular name of jatobá. It is a tree that demonstrates medicinal purposes and represents a food source in Brazil. The potential contribution of each species to recommended nutrient intakes (DRI) and Brazilian Health Surveillance Agency (ANVISA) for children and adults were calculated. A comparison between the quantified contents of micro- and macronutrients Al, Ca, Cr, Cu, Fe, K, Mg, Mn, Na, Ni, P, S, and Zn in leaves tea and tea of the bark of this plant was made with the recommendation of FDA and ANVISA. General safety regarding metal content (Na, K, Ca, Fe, Zn, and Ni) and nonmetal (P) was assured as none of them exceed the safety limit of the daily intake. However, the content of Mn observed in the teas of* H. martiana* Hayne is above the UL for tolerable intake level for children aged 1-6 years. There are no limits established for the UL of Cr and S for children and adults. The data on quantification of mineral concentration in the species* Hymenaea martiana *Hayne obtained can serve as a tool to decide the dosage of preparations from this plant used for medicinal purposes.

## 1. Introduction

The use of medicinal plants have always been important as an alternative source of medicine from early days until today, beyond being used as a source of food and in cultural representation [1]. The Cerrado region in Brazil is very rich and has great agricultural potential and is considered a source of varied species with high Brazilian consumption, whether in nature or for the treatment of diseases. Among the species found in Brazil, there is a tree known as Jatobá (*Hymenaea martiana* Hayne) that has pharmaceutical purposes [2], antioxidant activity [3], microbial, antiviral, hepatoprotective, gastroprotective [4], and antifungals [5]. In Brazil and in other countries, some species of the genus* Hymenaea* (Fabaceae) are used in folk medicine to treat diarrhea, dysentery, intestinal cramps, and other diseases [6].

Medicinal plants have therapeutic properties and are nutritionally important because of their mineral contents. In fact, macro- and microelements are requirements for promoting physical growth, sexual maturation, neuromotor development, and for the integrity and function of the immune system [7]. On the other hand, heavy metals can accumulate in human body over a long period and may cause adverse effects on human health; therefore, it is important to have a look on good quality control of medicinal plants in order to protect consumers from contamination by ingestion.

According to World Health Organization (WHO) [8], children are more susceptible to heavy metals than adults. The diagnosis of a case of lack or toxicity reveals a functional or physiological alteration. Factors such as age and gender are the main influencers of clinical symptoms whose diagnosis is usually made in a more advanced state of the pathology [9].

Concentration of macro- and microelements in the leaves, leaves tea, bark, and tea bark of Jatobá were determined by inductively coupled plasma optical emission spectrometry (ICP OES) [10]; however, there are no studies on the contents of the tea from leaves and barks of this plant compared to value of dietary reference intake (DRI). The aim of the present study was to report the content of the bark tea and leaf tea of* Hymenaea martiana* Hayne in relation to the tolerable levels of maximum intake for children and adults based on the FDA and National Agency for Sanitary Surveillance (ANVISA). The permissible limits of essential elements and toxic metal for medicinal plants have not yet been set by Brazil. Thus, the comparative data of our studies have relevant information for the use of medicinal plants that can be included in the data on the plant species included in the Renisus (National Relation of Medicinal Plants of Interest to SUS).

## 2. Materials and Methods

### 2.1. Plant Material

**Figure 1:**
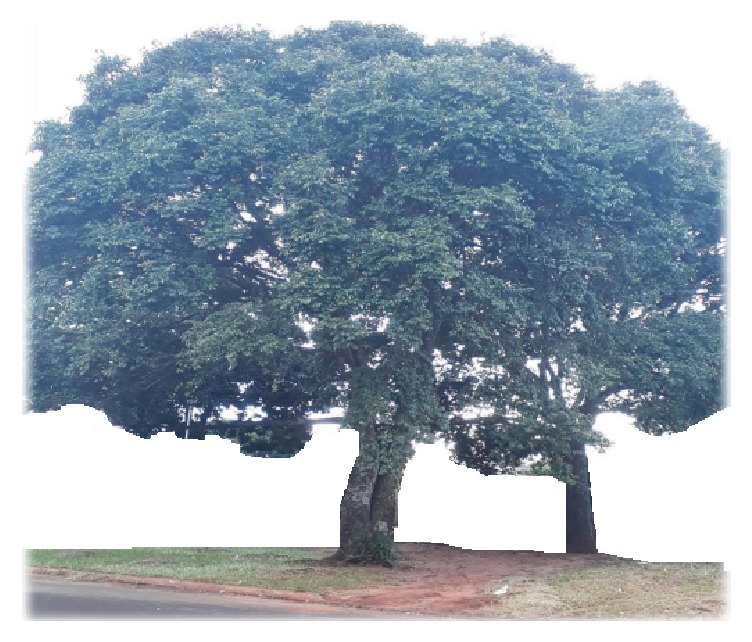
*Hymenaea martiana *Hayne–Fabaceae (jatobá) (photo: Layza S Rocha and Valter A Nascimento).

Plant material (leaves and barks of* Hymenaea martiana *Hayne) was collected in an urban area of the city of Campo Grande, Mato Grosso do Sul, Brazil (Figure 1). The plant was deposited in the herbarium of Federal University of Mato Grosso do Sul (Brazil) and authenticated by the biologist Dr. Flávio Macedo Alves, exsiccatae number: 64779 CGMS (H. martiana Hayne-Fabaceae). The project was registered in the National System of Genetic Resource Management and Associated Traditional Knowledge (SisGen, n° A7716EC).

*Hymenaea* L., belonging to the family Fabaceae (Caesalpinioideae), is of tropical climate, with a trunk with resinous exudate and bifoliolated leaves. The flowers are whitish, arranged in axillary and terminal racemes. Woody fruit with brown pericarp, indehiscent, contains seeds surrounded by yellow flesh. It flowers from December to January and has its ripe fruits from August to September. In Brazil, it is popularly called jatobá, jatobá-do-campo, jatobá-da-serra, jatobá-capão, jatobá-de-casca-fina, or jataí [11]. It is a tree (Figure 1) up to 10-20 meters high, with trunk diameter of 30-70 centimeters, whose fruits are used as food, and teas from leaves and barks have medicinal utility—Botany Synonymy:* Hymenaea chapadensis* Barb. Rodr.,* Hymenaea correana* Barb. Rodr.* Hymenaea olfersiana* Hayne, and* Hymenaea rotundata* Hayne [12].

### 2.2. Preparation of Medicinal Plant: Digestion Procedure

The barks of the plant samples were washed thoroughly in ultrapure water so as to remove superficial dust. The samples dried by placing it in an oven at a temperature of 50°C for a period of 48 hours until the stabilization of their weights. The dried samples were ground to a fine powder using a stainless steel grinder.

Infusions were prepared considering the recommended proportion for consumption: 400 grams of fresh bark was weighed into glass beakers of 250 mL. Boiling ultra-pure water was added to the bark and kept for 15 min covered with a watch glass to extract sample components. After cooling, the tea infusion was filtered to 50 mL volumetric flasks, acidified with 5 ml of concentrated HNO_3_ (65% Merck), and the final volume adjusted to 30 mL with ultrapure water (Millipore, Milli-Q Biocel Water Purification System, Germany). All analyses were carried out in triplicate and analytical blanks were also prepared following the same procedure used for the samples. Elemental analysis by the technique of Inductively Coupled Plasma Optical Emission Spectrometry (ICP OES) and all calibration parameters of ICP OES curves were published according to [10].

## 3. Results and Discussion

The values of daily food intake stipulated by the Brazilian National Health Surveillance Agency (ANVISA) are the same as those of the Dietary Intakes Reference (DRI) of the guidelines prepared by the Office of Nutrition and Food Labelling of the Center for Food Safety and Applied Nutrition at the US Food and Drug Administration (FDA) in 2016. According to Brazilian ANVISA guide, the quantified elements are considered as a source when it presents at least 15% of DRI and high content when it represents at least 30% of DRI per serving or mg/100g. Therefore, based on the Brazilian ANVISA, it is possible to determine if the leaves tea or bark tea of Jatobá has minimal nutritional elemental content or if they can cause adverse health effects in the Brazilian population.

*Dietary reference intake values vary by age, gender, and stage of life of healthy people *[13]*. The reference values include the following: the estimated average requirement (EAR) is the intake level for a nutrient at which the needs of 50% of the population will be met; *recommended dietary allowance (RDA): average daily level of intake sufficient to meet the nutrient requirements of nearly all healthy people (98%); adequate intake (AI): established when evidence is insufficient to develop an RDA and is set at a level assumed to ensure nutritional adequacy; tolerable upper intake level (UL): maximum daily intake unlikely to cause adverse health effects.

The results for the mean concentration of studied contents in herbal infusions are presented in Tables 1 and 2. From concentration of the content found in the bark tea and leaves tea of Jatobá were calculated the percentages values stipulated by RDI according to FDA and ANVISA for children and adults.

According to the calculations of percentages obtained from the stipulated intake values by ANVISA (Tables 1 and 2), it is observed that bark tea and leaves tea are not sources of Na, K, Ca, Fe, and P for children and adults. Bark tea is a source of Mg for children aged 1 to 6 years and is not source of this element for children 7 to 10 years of age and adults according to ANVISA (Table 1). In addition, leaves tea bare high content of Mg for children (1-3 y) and source of this element for children aged 4-10 years. The leaves teas are not source of Mg for adults (Table 2).

The bark tea and leaves tea of* H. martiana *Hayne are source of Zn for children aged 1 to 6 years but are not sources for children aged 7-10 years and adults. There is no RDI for consuming S and Ni in children and adults. The concentrations of Mn and Cr in bark tea and leaves tea were compared with the RDA, and it was concluded that those teas have a high content of Mn and Cr for children and adults (Tables 1 and 2).

The contents of Na, Ca, Mg, P, Fe, Zn, and Ni in the teas of* H. martiana *Hayne are below the UL for tolerable intake level for children and adults (Tables 1 and 2). From comparison, the content of elements Na, Ca, Mg, P, Fe, Zn, and Ni in teas of Jatobá does not represent a risk of adverse health effects for children and adults. However, the effects of exposure to any hazardous substance and especially medicinal plants depend on dose, duration, and whether other chemicals are present.

According to a clinical case, only four tablespoons of rock salt leads to acute toxicity in a six-year-old child [14]. In addition, Calcium (Ca) can reduce the absorption of Antibiotics of the fluoroquinolone and tetracycline families when taken together [15]. On the other hand, ingestion of large quantities of magnesium through food does not pose a health risk to healthy individuals because the kidneys eliminate excessive amounts in the urine [16].

Iron toxicity from food sources is rare. In the United States, medications and supplements were the leading cause of iron poisoning and were a leading cause of death from unintentional poisoning in children under 6 years of age [17].

There are no reports of chronic or long-term food poisoning due to oral zinc intake. However, toxic effects associated with consumption of Zinc by Dietary supplements have been reported in the last years [18].

In Tables 1 and 2, the concentration of nickel detected in bark tea and leaf tea was 0.05 mg/100 g and 0.092 mg/100 g. The concentration of nickel in the leaves tea of* H. martiana *Hayne is higher than many food products having a nickel content of less than 0.05 mg/100 g fresh weight. Nickel and its compounds are carcinogenic. However, in humans, most of the nickel ingested in the diet is not absorbed, being eliminated in feces and urine [19], milk [20], by sweat [21], with saliva [22], and hair [23].

The UL for potassium in children and adults has not been established. WHO recommends an increase in potassium intake from food to control blood pressure in children and adults [24]. Excess amounts of potassium are normally excreted from the body [25].There are no reports of toxicity of potassium from consumption in food and medicinal plants. Thus, potassium does not represent a risk of adverse health effects for children and adults.

There is not tolerable upper intake level (UL) for consuming of Sulfer in children and adults. We were unable to find studies involving the toxicity of sulfur by ingestion of food or medicinal plants. Many simple sulphur derivates are toxic, such as sulphur dioxide (SO_2_) and hydrogen sulfide. Bloating or flatulence is possible adverse effects due to the consumption of foods containing sulfur or supplements containing sulfur in higher amounts [26].

The content of Mn observed in the teas of* H. martiana* Hayne is above the UL for tolerable intake level for children aged 1-6 years. Manganese (Mn) acts as an activator of enzymes and components of metalloenzymes. However, as for its toxicity, this element may affect the cerebral and may lead to a neurological syndrome similar to Parkinson's [27]. Children have higher intestinal absorption of manganese, as well as lower biliary excretion of manganese when compared to adults [28]. In fact, children are susceptible to any negative, neurotoxic effects of manganese. Studies during years have raised concern over the deleterious cognitive and behavioral effects of manganese exposure in children [29].

The UL limits have not been set yet for chromium for children and adults. However, chromium (III) and chromium (VI) are toxic. Clinical evidence reports the death of an adolescent 14 years of age after ingestion of 7.5 mg/kg of potassium dichromate. Before death, the patient presented with severe abdominal pain and vomiting. High levels of liver enzymes were found in the serum after 24 hours of ingestion, and exams showed necrosis of the liver and kidneys [30].

In addition, there is the relation of the case of two people who ingested oats contaminated with potassium dichromate and presented acute signs and symptoms of intoxication, such as abdominal pain, vomiting, and diarrhea [30]. There are insufficient data to establish a safe upper limit for chromium intake. In addition, children and adults should take special care with respect to the ingestion of* H. martiana *Hayne in large quantities. There are no limits established by the Brazin government on intake of this plant.

## 4. Conclusion

The teas from leaves and bark of* H. martiana *Hayne contain mineral elements that have nutritional value for children and adults; further research using animal models is required to determine the availability of the nutrients and the effects of processing on chemical and nutritive values of the plant.

The results of this study indicate a low accumulation of metals such as Na, K, Ca, Fe, Zn, Ni, and nonmetal (P) in the plant, which is relevant for the evaluation of the quality of its safe use as a phytotherapeutic potential.

The content of Mn observed in the teas of* Hymenaea martiana *Hayne is above the UL for tolerable intake level for children aged 1-6 years. The UL limits have not yet been set for Cr and S for children and adults. The data obtained in this study on contents of Jatobá can be useful in deciding the dosage of the drugs prepared from the plant.

## Figures and Tables

**Table 1:** Comparison of the concentration of Jatobá-*Hymenaea martiana *Hayne (*Fabaceae*) bark tea (mg/100 g) with the RDA/AI*∗* and UL (mg/day) established for children and adults by FDA and ANVISA/Brazil.

	Bark tea (mg/100 g)	Children 1-3 years (mg/day)			Children 4-6 years (mg/day)			Children 7-10 years (mg/day)			Adults (mg/day)		
*Macro* ^*#*^		*RDA/AI∗*	*%*	*UL*	*RDA/AI∗*	*%*	*UL*	*RDA/AI∗*	*%*	*UL*	* RDA/AI∗*	*%*	*UL*
Na	13.46	1000*∗*	1.34	1500	120*∗*	1.12	1900	1500*∗*	0.897	2200	1500*∗*	0.89	2300
K	199.49	3000*∗*	6.64	ND	380*∗*	5.24	ND	4500*∗*	4.43	ND	4700*∗*	4.24	ND
Ca	25.59	500	5.11	2500	600	4.26	2500	700	3.65	3000	1000	2.55	2666
Mg	11.18	60	18.63	65	73	15.31	350	100	11.18	350	260	4.3	350
S	58.7	ND	ND	ND	ND	ND	ND	ND	ND	ND	ND	ND	ND
P	20.26	460	4.40	3000	500	4.05	3000	1250	1.62	4000	700	2.89	3500
*Micro* ^*#*^													
Fe	0.19	6	3.31	40	6	3.31	40	9	2.21	40	14	1.42	45
Zn	1	4.1	20.24	7	5.1	16.27	12	5.6	14.82	23	7	11.85	38
Ni	0.05	ND	ND	0.2	ND	ND	0.3	ND	ND	0.6	ND	ND	1
Mn	3.03	1.2*∗*	252.7	2	1.5*∗*	202.3	3	1.5*∗*	202.3	6	2.3*∗*	131.1	10.3
Cr	0.188	0,011	1709	ND	0.015	1253	ND	0.015	1253	ND	0.035	537.1	ND

^#^Data published in Ref. [10]; *∗*Adequate Intake (AI); ND: Not determinable

**Table 2:** Comparison of the concentration of Jatobá-*Hymenaea* martiana Hayne (*Fabaceae*) leaves tea (mg/100 g) with the RDA/AI*∗* and UL (mg/day) established for children and adults by FDA and ANVISA/Brazil.

	Leaves tea (mg / 100 g)	Children 1-3 years (mg/day)			Children 4-6 years (mg/day)			Children 7-10 years (mg/day)			Adults (mg/day)		
*Macro* ^*#*^		*RDA/AI∗*	*%*	*UL*	*RDA/AI∗*	*%*	*UL*	*RDA/AI∗*	*%*	*UL*	*RDA/AI∗*	*%*	*UL*
Na	15.17	1000*∗*	1.51	1500	1200*∗*	1.26	1900	1500*∗*	1.011	2200	1500*∗*	1.01	2300
K	280.02	3000*∗*	9.33	ND	3800*∗*	7.36	ND	4500*∗*	6.22	ND	4700*∗*	5.95	ND
Ca	19.72	500	3.94	2500	600	3.28	2500	700	2.81	3000	1000	1.97	2666
Mg	20.93	60	34.88	65	73	28.67	350	100	20.93	350	260	8.05	350
S	26.07	ND	ND	ND	ND	ND	ND	ND	ND	ND	ND	ND	ND
P	34.3	460	7.45	3000	500	6.86	3000	1250	2.74	4000	700	4.9	3500
*Micro* ^*#*^													
Zn	0.794	4.1	19.36	7	5.1	15.56	12	5.6	14.17	23	7	11.34	38
Ni	0.092	ND	ND	0.2	ND	ND	0.3	ND	ND	0.6	ND	ND	1
Mn	3.6	1.2*∗*	300	2	1.5*∗*	240	3	1.5*∗*	240	6	2.3*∗*	156.52	10.3
Cr	0.100	0.011	909	ND	0.015	666.6	ND	0.015	666.6	ND	0.035	285.7	ND

^#^Data published in Ref. [10]; *∗*Adequate Intake (AI); ND: Not determinable

## Data Availability

The [concentration of macro- and micronutrients in the leaves, leaves tea, bark and bark tea of Jatobá] data used to support the findings of this study are included and referenced within the article. In addition, readers can access the published article: L. S. Rocha, D. A. Gonçalves, D. G. Arakaki, P. F. S. Tschinkel, N. V. de Lima, L. C. S. de Oliveira, R. C. A. Guimarães, V. A. Nascimento, data on elemental composition of the medicinal plant Hymenaea martiana Hayne (Jatobá), Data in Brief, 19(2018), pp. 959-964. doi: 10.1016/j.dib.2018.05.142.
